# Targeting macrophage scavenger receptor 1 promotes insulin resistance in obese male mice

**DOI:** 10.14814/phy2.13930

**Published:** 2018-11-28

**Authors:** Joseph F. Cavallari, Fernando F. Anhê, Kevin P. Foley, Emmanuel Denou, Rebecca W. Chan, Dawn M. E. Bowdish, Jonathan D. Schertzer

**Affiliations:** ^1^ Department of Biochemistry and Biomedical Sciences Farncombe Family Digestive Health Research Institute Hamilton Ontario Canada; ^2^ Department of Pathology and Molecular Medicine and McMaster Immunology Research Centre McMaster University and Michael G. DeGroote Institute for Infectious Disease Research Hamilton Ontario Canada

**Keywords:** glucose, immunometabolism, inflammation, insulin, obesity

## Abstract

Immune components can bridge inflammatory triggers to metabolic dysfunction. Scavenger receptors sense lipoproteins, but it is not clear how different scavenger receptors alter carbohydrate metabolism during obesity. Macrophage scavenger receptor 1 (MSR1) and macrophage receptor with collagenous structure (MARCO) are scavenger receptors that have been implicated in lipoprotein metabolism and cardiovascular disease. We assessed glucose control, tissue‐specific insulin sensitivity, and inflammation in *Msr1*‐ and *Marco*‐deficient mice fed with obesogenic diets. Compared to wild‐type (WT) mice, *Msr1* ^−/−^ mice had worse blood glucose control that was only revealed after diet‐induced obesity, not in lean mice. Obese *Msr1* ^−/−^ mice had worse insulin‐stimulated glucose uptake in the adipose tissue, which occurred in the absence of overt differences in adipose inflammation compared to obese WT mice. *Msr1* deletion worsened dysglycemia independently from bacterial cell wall insulin sensitizers, such as muramyl dipeptide. MARCO was dispensable for glycemic control in obese mice. Oral administration of the polysaccharide fucoidan worsened glucose control in obese WT mice, but fucoidan had no effect on glycemia in obese *Msr1* ^−/−^ mice. Therefore, MSR1 is a scavenger receptor responsible for changes in glucose control in response to the environmental ligand fucoidan. Given the interest in dietary supplements and natural products reducing inflammation or insulin resistance in metabolic disease during obesity, our results highlight the importance of understanding which ligand–receptor relationships promote versus those that protect against metabolic disease factors. Our results show that ligand or gene targeting of MSR1 exacerbates insulin resistance in obese mice.

## Introduction

Low‐grade chronic inflammation can contribute to aspects of the metabolic syndrome, including altered endocrine control of metabolism. Inflammation can reduce the ability of insulin to alter carbohydrate metabolism in tissues that can lower blood glucose, which is often called insulin resistance (Hotamisligil et al. 1993, 1995). Higher levels of circulating and tissue‐resident cytokines, chemokines, and proinflammatory immune cells are typically associated with tissue insulin resistance, which is a factor that predicts and participates in whole body dysglycemia (Hotamisligil et al. 1993, 1995; Wellen and Hotamisligil 2005). For example, increased numbers of adipose tissue‐resident macrophages and inflammatory cytokines coincide with obesity‐related adipose tissue expansion (Hotamisligil et al. 1993; Weisberg et al. 2003). Furthermore, increased adipose‐resident macrophages are polarized to an inflammatory phenotype during obesity and adipose‐resident macrophages that are skewed toward proinflammatory characteristics correlate with body mass index (BMI) and indices of insulin resistance (Weisberg et al. 2003; Xu et al. 2003).

Innate and adaptive immune responses in the adipose tissue and the intestine have been shown to connect inflammation with insulin resistance (Winer and Winer 2012; McPhee and Schertzer 2015; Winer et al. 2016). Many sources of this metabolic inflammation have been characterized, including microbial or dietary components (Cani et al. 2007; Oliveira et al. 2013; Chi et al. 2014; Henriksbo et al. 2014; Caesar et al. 2015), endogenous metabolites (Mills et al. 2016; Liu et al. 2017), xenobiotics (Pestana et al. 2017), and therapeutic drugs (Henriksbo et al. 2014; Henriksbo and Schertzer 2015). Pattern recognition receptors (PRR) can bridge potential triggers of inflammation to metabolic outcomes by acting as sensors of pathogen‐associated molecular patterns (PAMPs) and/or damage‐associated molecular patterns (DAMPs). There are many examples of PRRs propagating metabolic inflammation and promoting insulin resistance and defects in carbohydrate metabolism during aging, obesity, or other stressors (Shi et al. 2006; Schertzer et al. 2011; Vandanmagsar et al. 2011; Henriksbo et al. 2014; Bauernfeind et al. 2016; McBride et al. 2017). However, some PRRs protect against metabolic inflammation and insulin resistance during obesity (Denou et al. 2015; Cavallari et al. 2017). It has now been shown that PRRs can reprogram cellular metabolism and propagate inflammation as opposed to being direct sensors for obesity‐associated inflammatory “ligands”, such as saturated fatty acids (Lancaster et al. 2018). There is still much to learn about how obesity‐related triggers of inflammation engage elements of the immune system to alter cellular and systemic metabolism.

PRRs can respond to ingested nutrients and scavenger receptors are well known as receptors for various lipoproteins (Parthasarathy et al. 1986; Babitt et al. 1997; Febbraio et al. 1999). For example, the Class B scavenger receptor SCARB1 has been implicated in lipoprotein metabolism and the Class B scavenger receptor SCARB3/CD36 has been implicated in long‐chain fatty acid metabolism (Babitt et al. 1997; Febbraio et al. 1999). Class A scavenger receptors have shown to detect and respond to modified low‐density lipoproteins (mLDL) (Parthasarathy et al. 1986). It is clear that different scavenger receptors are involved in macrophage foam cell formation, atherosclerosis, and cardiovascular disease (Ben et al. 2015; Zani et al. 2015), but the role of these scavenger receptors in glucose metabolism is not as well defined.

Macrophage scavenger receptor 1 (MSR1) and macrophage receptor with collagenous structure (MARCO) are Class A scavenger receptors and these PRRs are predominantly expressed in macrophages (Bowdish and Gordon 2009). MSR1 and MARCO sense lipoproteins that have been implicated in cardiovascular disease, but the roles for different Class A scavenger receptors in carbohydrate metabolism and insulin resistance are ill‐defined. Despite the connection between cardiovascular disease and diabetes, the roles of these scavenger receptors may be distinct to glucose metabolism. It is known that deletion of *Msr1* attenuates macrophage uptake of mLDL and limits atherosclerotic lesions in mice prone to atherosclerosis (Suzuki et al. 1997). Conversely, obese *Msr1*‐deficient mice display exacerbated insulin resistance and augmented inflammation characterized by polarization of macrophage populations toward more inflammatory subsets (Zhu et al. 2014). Less is known about the role of MARCO in obesity and insulin resistance and it is not clear if MARCO has a similar protective role in obesity‐induced insulin resistance. This is worth testing since MARCO has been shown to be necessary for both toll‐like receptor 2 (TLR2) and nucleotide oligomerization domain 2 (NOD2)‐mediated bacterial pathogen sensing and clearance (Dorrington et al. 2013). Deletion of *Tlr2* can protect against insulin resistance, whereas deletion of *Nod2* can exacerbate obesity‐induced insulin resistance in obese mice (Ehses et al. 2010; Denou et al. 2015). Furthermore, specific bacterial cell wall components, such as muramyl dipeptide (MDP) are NOD2‐dependent insulin sensitizers (Cavallari et al. 2017). These results warrant testing how MARCO alters insulin sensitivity and blood glucose control in comparison to other Class A scavenger receptors, such as MSR1.

In this study, we used *Msr1*‐ and *Marco*‐deficient mice fed obesogenic diets to assess the role of these scavenger receptors in regulating glucose control, tissue‐specific insulin sensitivity, and inflammation. We demonstrate that MARCO is dispensable for glycemic control in obese mice. Genetic deletion of *Msr1* or feeding fucoidan, a natural product ligand of MSR1, both worsened insulin resistance and blood glucose control. We found that poor blood glucose control coincided with impaired insulin‐stimulated glucose uptake in the adipose tissue, which occurred in the absence of overt adipose inflammation in obese *Msr1* ^−/−^ mice.

## Materials and Methods

### Animals and diets

All animal procedures were approved by the Animal Research Ethics Board of McMaster University and performed according to institutional guidelines. All mice were male and were maintained on a 12‐h light/dark cycle. Wild‐type (WT) C57BL/6J mice were from The Jackson Laboratory (Cat# 000664). *Msr1* ^−/−^ mice on a C57BL/6J background were originally from the laboratory of S. Gordon (Suzuki et al. 1997). *Marco* ^−/−^ mice on a C57BL/6N background were originally from the laboratory of K. Tryggvason (Arredouani et al. 2004). For all studies, mice were 9–10 weeks old prior to starting experiments or switching diets. Mice were fed a control diet (17% kcal from fat, 29% kcal from protein, 54% kcal from carbohydrate; Cat# 8640 Teklad 22/5; Envigo, Huntington, United Kingdom) or an obesogenic, high‐fat, low‐fiber diet (60% kcal from fat, 20% kcal from protein, 20% kcal from carbohydrate; Cat# D12492; Research Diets, New Brunswick, NJ, USA) as indicated for each experiment. Our study was designed to test glycemic perturbations of an obesogenic diet that is higher in fat and lower in fiber compared to a standard rodent diet. Muramyl dipeptide (MDP; cat# tlrl‐mdp; Invivogen, San Diego, CA, USA) was administered via intraperitoneal injection at a dose of 100 *μ*g/mouse for 3 days prior to metabolic tests. Fucoidan from *Fucus vesiculosus* (Cat# F5631; MilliporeSigma, Burlington, MA, USA) was administered by oral gavage at a dose of 40 mg/kg three times per week for 4 weeks.

### Genotyping

Mouse liver was digested in buffer containing 100 mmol/L Tris‐HCl, 5 mmol/L ethylenediaminetetraacetic acid, 200 mmol/L NaCl, 0.2% w/v SDS, and 1.5 units of proteinase K (Cat# EO0491; Thermo Fisher Scientific, Waltham, MA, USA) at 37°C overnight. DNA was precipitated from tissue lysate by adding an equal volume of isopropanol and gently agitating the mixture. DNA pellets were washed twice with a 75% ethanol/25% ultrapure water solution and suspended in ultrapure water.

PCR amplification of isolated DNA was performed using primer sequences targeting *Msr1* and *Marco* genes. *Msr1* was amplified from DNA samples with the following primer sequences: “WT Forward” ACC TTA TAG ACA CGG GAC GCT TCC AGA A, “WT Reverse” GAC TCT GAC ATG CAG TGT TTC TGT A, “KO Forward” ACC TTA TAG ACA CGG GAC GCT TCC AGA A, and “KO R Reverse” AGG AGT AGA AGG TGG CGC GAA GG. *Marco* was amplified from DNA samples with the following primer sequences: “WT Forward” CAG CTG GGT CCA TAC CAG C, “WT Reverse” CTG GAG AGC CTC GTT CAC C, “KO Forward” CCA CGC TCA TCG ATA ATT TCA C, and “KO Reverse” GCC TGC AGT GGC CGT CGT TTT A. Amplified sequences were separated on a 1% agarose gel.

### Glucose and insulin tolerance tests

Glucose tolerance tests (GTTs) and insulin tolerance tests (ITTs) were performed in 6 h fasted and conscious mice. D‐(+)‐Glucose (Cat# G7021; MilliporeSigma, Burlington, MA, USA) and insulin aspart (NovoRapid; Novo Nordisk, Bagsværd, Denmark) were delivered via intraperitoneal injection at doses indicated in figure legends. Blood glucose was determined by tail vein blood sampling at the indicated time points using a handheld glucometer (Accu‐Chek Performa; Roche, Basel, Switzerland).

### Glucose uptake and adiposity imaging

Insulin stimulated uptake of 2‐deoxy‐2‐(^18^F)fluoro‐D‐glucose into various tissues was measured by positron emission tomography as previously described (Jorgensen et al. 2013). Adiposity of these mice was determined by computed tomography as previously described (Cavallari et al. 2017).

### Gene expression

Total RNA was obtained from frozen mouse white adipose tissue via mechanical homogenization at 4.5 m/sec for 30 sec using a FastPrep‐24 tissue homogenizer (MP Biomedicals, Santa Ana, CA, USA) and ceramic beads, followed by guanidinium thiocyanate–phenol–chloroform extraction. RNA was treated with DNase I (Cat# 18068‐015; Thermo Fisher Scientific, Waltham, MA, USA) and cDNA was prepared using 1000 ng total RNA and SuperScript III Reverse Transcriptase (Cat# 18080‐044; Thermo Fisher Scientific, Waltham, MA, USA). Transcript expression was measured using TaqMan Assays with AmpliTaq Gold DNA polymerase (Cat# N8080247; Thermo Fisher Scientific, Waltham, MA, USA) in a Rotor‐Gene Q real‐time PCR cycler (QIAGEN, Hilden, Germany), and target genes were compared to the geometric mean of *Rplp0* and *Rn18s* housekeeping genes using the ΔΔ*C*_*T*_ method. Gene expression was analyzed in WT mice treated with vehicle (*n* = 7), WT mice treated with fucoidan (*n* = 5), SRA^−/−^ mice treated with vehicle (*n* = 6) and SRA^−/−^ mice treated with fucoidan (*n* = 8).

### Data analyses

Values are reported as mean ± standard error of the mean (SEM) and *P* < 0.05 was considered statistically significant. Comparisons of each result were analyzed by unpaired two‐tailed *t*‐test or one‐ or two‐way ANOVA with Tukey post hoc testing, as indicated. Nonparametric tests were utilized on data sets that were not normally distributed. GraphPad Prism 6 software was used. Values of n represent different mice for each experiment and are represented by symbols in the figures.

### Data availability

The datasets generated during and analyzed during this study are available from the corresponding author on reasonable request.

## Results

### Deletion of *Msr1*, but not *Marco*, worsened HFD‐induced insulin resistance

**Figure 1:**
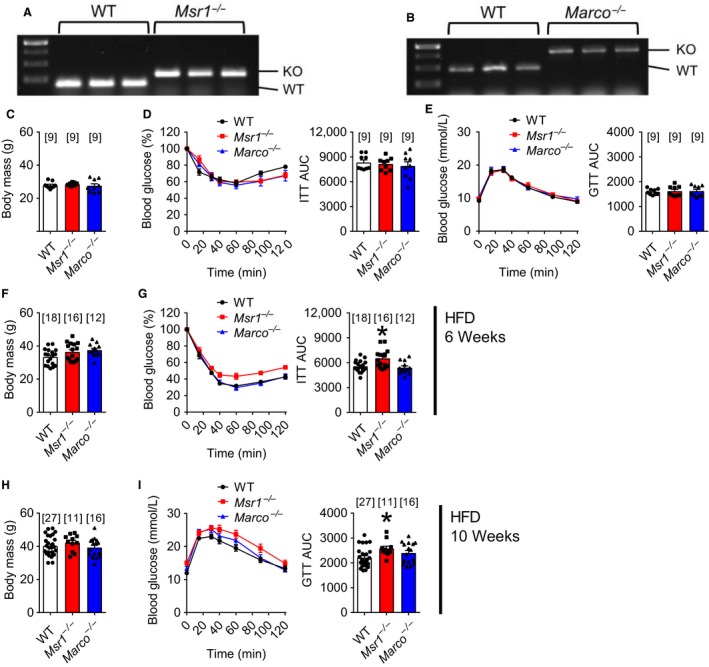
Deletion of macrophage scavenger receptor 1 (*Msr1*) worsens high‐fat diet‐induced glucose and insulin intolerance. Electrophoresis‐separated PCR amplification products showing the genotyping of WT and *Msr1* ^−/−^ mice (A), and WT and *Marco* ^−/−^ mice (B). Body mass (C), blood glucose, and cumulative area under curve (AUC) during an insulin tolerance test (0.5 IU/kg; D) and glucose tolerance test (2 g/kg; E) of WT,* Msr1* ^−/−^, and *Marco* ^−/−^ mice fed a control diet. Body mass (F) and blood glucose and cumulative AUC during an insulin tolerance test (1  IU/kg; G) of WT,* Msr1* ^−/−^, and *Marco* ^−/−^ mice fed a high‐fat diet (HFD) for 6 weeks. Body mass (H) and blood glucose and cumulative AUC during a glucose tolerance test (1 g/kg; I) of WT,* Msr1* ^−/−^, and *Marco* ^−/−^ mice fed a HFD for 10 weeks. Body mass and AUC graphs were analyzed by one‐way ANOVA with Tukey post hoc testing. Data are means ± SE. * indicates significance with *P* < 0.05. Numbers of mice analyzed for each condition are represented above each bar and by symbols.

We used mice that had a genetic deletion of *Msr1* or *Marco* to determine if different Class A Scavenger receptors were relevant to obesity‐induced insulin resistance (Fig. 1A and B). Wild‐type (WT), *Msr1* ^−/−^ and *Marco* ^−/−^ mice that were fed a control diet containing ~17% energy from fat had no difference in body mass or blood glucose levels during an insulin tolerance test (ITT) or glucose tolerance test (GTT) (Fig. 1C–E). We then showed that WT, *Msr1* ^−/−^, and *Marco* ^−/−^ mice all had similar body mass after 6 weeks of feeding an obesogenic, low‐fiber, high‐fat diet (HFD) (Fig. 1F). However, 6 weeks of HFD revealed that only *Msr1* ^−/−^ mice had higher blood glucose during an ITT compared to WT mice or *Marco* ^−/−^ mice (Fig. 1G; *P* = 0.0068). In further support of *Msr1* deletion worsening HFD‐induced insulin resistance, we found that 10 weeks of HFD‐feeding caused higher blood glucose during a GTT in *Msr1* ^−/−^ mice compared to WT and *Marco* ^−/−^ mice. (Fig. 1I; *P* = 0.0322). This glycemic effect was independent of changes in body mass between different genotypes of HFD‐fed mice (Fig. 1H). These results show that *Msr1* deletion worsens HFD‐induced insulin resistance, whereas MARCO is dispensable during diet‐induced obesity in mice.

### *Msr1* deletion worsens adipose tissue insulin resistance in obese mice

**Figure 2:**
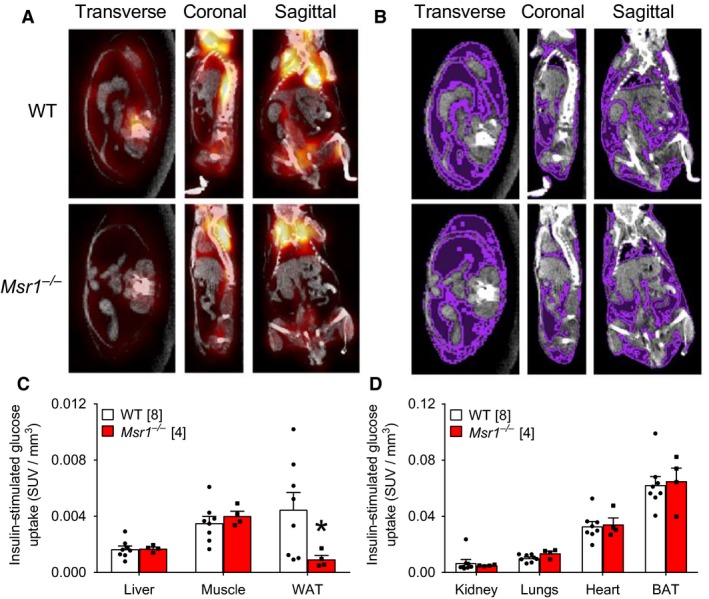
Deletion of macrophage scavenger receptor 1 (*Msr1*) worsens adipose tissue insulin sensitivity. Visualization of insulin‐stimulated 2‐deoxy‐2‐(^18^F)fluoro‐D‐glucose uptake (orange; A) and adipose tissue (purple, determined by computed tomography; B) in WT and *Msr* ^−/−^ mice fed a high‐fat diet (HFD). Quantification of insulin‐stimulated 2‐deoxy‐2‐(^18^F)fluoro‐D‐glucose uptake in key insulin‐responsive metabolic tissues (C) and other tissues (D) as measured by positron emission tomography of WT and *Msr1* ^−/−^ mice fed a high‐fat diet. Data was analyzed by unpaired two‐tailed *t*‐test. Data are means ± SE. * indicates significance with *P* < 0.05. Numbers of mice analyzed for each condition are represented by symbols.

We next used radiolabeled glucose tracer and whole‐body imaging after insulin injection in 6‐week HFD‐fed WT and *Msr1* ^−/−^ mice in order to determine tissue‐specific insulin resistance (Fig. 2A and B). We found that insulin‐stimulated glucose uptake was lower in the white adipose tissue (WAT) of HFD‐fed *Msr1* ^−/−^ mice compared to WT mice (Fig. 2C; *P* = 0.0485). We found that *Msr1* ^−/−^ mice and WT mice had similar insulin stimulated glucose uptake in all other tissues that were analyzed, including liver, skeletal muscle, heart, kidney, lungs, and brown adipose tissue (Fig. 2C and D). These results show that *Msr1* deletion worsens WAT insulin resistance during diet‐induced obesity in mice.

### Bacterial insulin sensitizers lower glucose independent of MSR1 in obese mice

**Figure 3:**
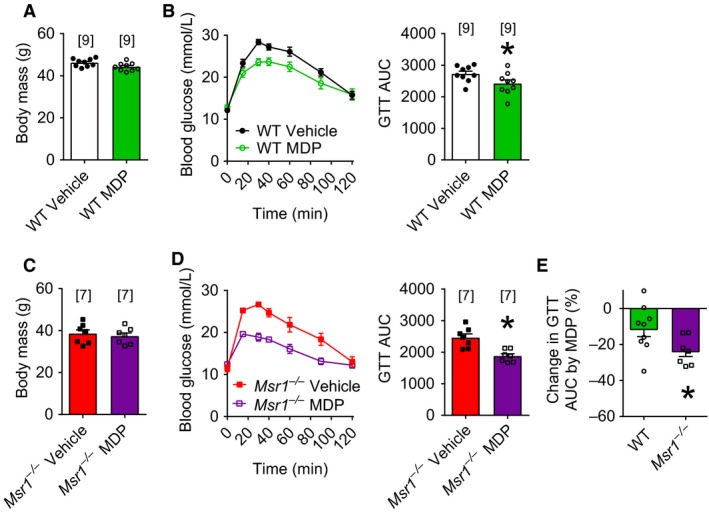
The postbiotic muramyl dipeptide (MDP) improves glycemia independent of macrophage scavenger receptor 1 (*Msr1*). Rather, muramyl dipeptide improvements in glucose tolerance are potentiated by deletion of *Msr1*. Body mass (A) and blood glucose and cumulative area under curve (AUC) during a glucose tolerance test (1 g/kg; B) of WT mice fed a high‐fat diet for 16 weeks and treated with saline or MDP for 3 days. Body mass (C) and blood glucose and cumulative AUC during a glucose tolerance test (1 g/kg; D) of *Msr1* ^−/−^ mice fed a high‐fat diet for 16 weeks and treated with saline or MDP for 3 days. Body mass and AUC graphs were analyzed by unpaired two‐tailed *t*‐tests. Data are means ± SE. * indicates significance with *P* < 0.05. Numbers of mice analyzed for each condition are represented above each bar and by symbols.

We next hypothesized that *Msr1* ^−/−^ mice had worse insulin resistance because of defective bacterial cell wall muropeptide/peptidoglycan sensing, similar to *Nod2* ^−/−^ mice (Denou et al. 2015). We therefore tested how glycemic control was altered by MDP, a bacterial cell wall muropeptide known to promote insulin sensitivity via NOD2 (Cavallari et al. 2017). We found that MDP treatment lowered glucose during a GTT in both WT (Fig. 3A and B; *P* = 0.0499) and *Msr1* ^−/−^ (Fig. 3C and D; *P* = 0.0014) mice without changing the body mass of obese mice fed a HFD for 16 weeks. We found that MDP lowered the cumulative area under the curve for glucose during a GTT to a greater extent in *Msr1* ^−/−^ compared to WT mice (Fig. 3E; *P* = 0.048). These results show that MDP improves glycemia independent of MSR1. Deletion of *Msr1* actually potentiates glucose clearance in response to the NOD2 ligand, MDP.

### The polysaccharide fucoidan worsens insulin resistance via MSR1 in obese mice

**Figure 4:**
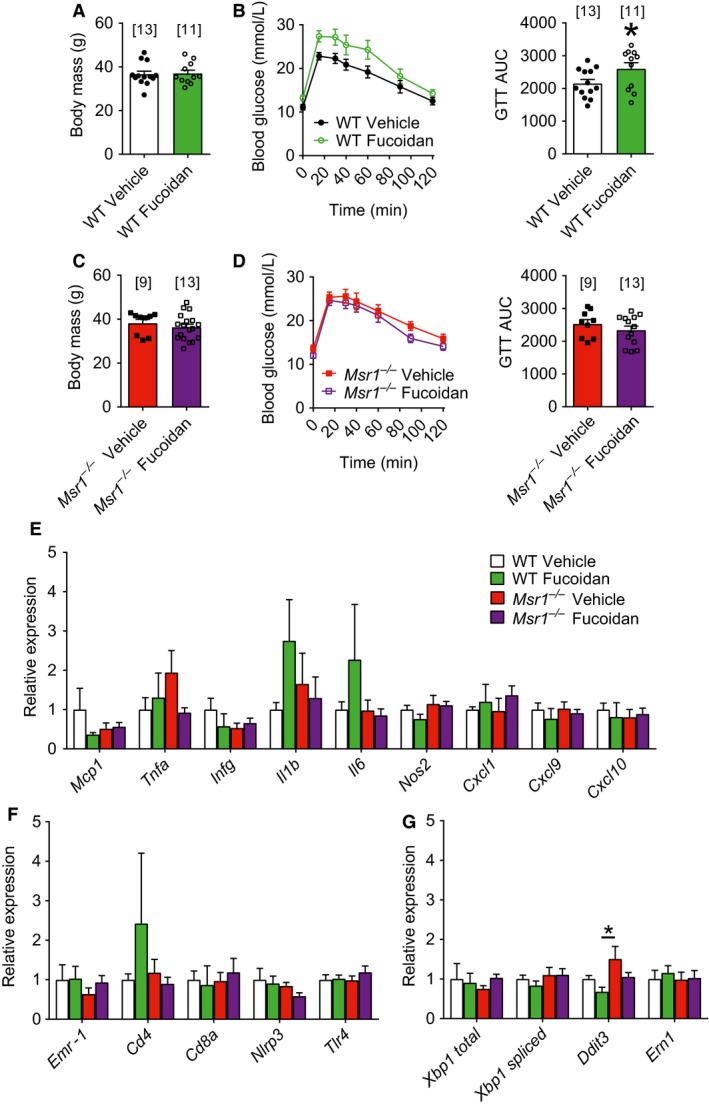
Fucoidan‐mediated worsening of glucose tolerance during obesity requires functional macrophage scavenger receptor 1. Body mass (A) and blood glucose and cumulative area under curve (AUC) during a glucose tolerance test (1.5 g/kg; B) of WT mice fed a high‐fat diet for 4 weeks and treated with vehicle or fucoidan three times per week for 4 weeks. Body mass (C) and blood glucose and cumulative AUC during a glucose tolerance test (1.5 g/kg; D) of *Msr1* ^−/−^ mice fed a high‐fat diet for 4 weeks and treated with vehicle or fucoidan three times per week for 4 weeks. Transcript levels of cytokines and chemokines (E), immune cell receptors (F), and endoplasmic reticulum stress markers (G) of WT and *Msr1* ^−/−^ mice fed a high‐fat diet for 4 weeks and treated with vehicle or fucoidan three times per week for 4 weeks. Body mass and AUC graphs were analyzed by unpaired two‐tailed *t*‐tests. Transcript levels were analyzed by two‐way ANOVA with Tukey post hoc testing. Data are means ± SE. * indicates significance with *P* < 0.05. Numbers of mice analyzed for each condition are represented above each bar and by symbols.

We next tested if a suspected ligand for MSR1 would alter glucose control. We found that oral delivery of fucoidan (40 mg/kg body mass, three times per week for 4 weeks) caused higher blood glucose during a GTT in WT mice fed a HFD for 4 weeks despite no change in body mass (Fig. 4A and B; *P* = 0.0494). We next demonstrated the importance of MSR1 in fucoidan's effect on glycemia, since oral delivery of fucoidan did not change body mass or blood glucose during a GTT in *Msr1* ^−/−^ mice fed a HFD for 4 weeks (Fig. 4C and D). These data show that engaging MSR1 with the polysaccharide fucoidan worsens glucose control in an MSR1‐dependent manner without changing body mass. We also tested if genetic deletion of *Msr1* or natural product targeting of MSR1 altered adipose tissue inflammation. We hypothesized that adipose tissue inflammation could underpin adipose insulin resistance observed in HFD‐fed fucoidan‐treated WT mice or *Msr1* ^−/−^ mice. We found that neither fucoidan‐treated mice nor *Msr1* ^−/−^ mice showed overt signs of inflammation, as suggested by no differences in transcript levels of inflammatory cytokines, chemokines, immune cell markers, or ER stress markers (Fig. 4E–G).

## Discussion

Components of the immune system such as PRRs can link inflammation to metabolic dysfunction. We sought to understand if different Class A scavenger receptors influenced blood glucose control during diet‐induced obesity in mice. We found that genetic deletion of *Msr1* exacerbated adipose tissue insulin resistance. These results further reinforce the concept that not all immune components or PRRs promote metabolic inflammation and insulin resistance. We add further evidence that loss of *Msr1* worsens insulin resistance during obesity, which is consistent with previous reports showing that *Msr1* (i.e., SR‐A) deletion deteriorates adipose tissue insulin sensitivity in obese mice (Zhu et al. 2014). Our results build on this previous work by showing that MSR1 provides a unique protection from excessive insulin resistance compared to other Class A scavenger receptors, since MARCO was dispensable for changes in insulin sensitivity in obese mice.

*MSR1* transcript levels in the adipose tissue of humans showed stronger correlation with insulin sensitivity compared to SCARB3/CD36, a Class B Scavenger receptor that has been intensely studied at the nexus of lipid metabolism and insulin resistance (Goudriaan et al. 2003; Elbein et al. 2011; Wilson et al. 2016). Given that *MSR1* transcript levels are increased, mainly in the stromal vascular fraction of adipose tissue of insulin‐resistant subjects, it is important to consider if this association represents a potential cause or protective response from excessive insulin resistance. Hyperinsulinemia is a key factor in driving insulin resistance and it is already known that insulin lowers *Msr1* levels in human macrophages (Park et al. 2012). Hence, our data showing that *Msr1* deletion exacerbates insulin resistance in adipose tissue of obese mice is consistent with a model where MSR1 acts as a compensatory or protective response to limit excessive insulin resistance. It is not yet clear what stimuli underpin changes in MSR1 levels during obesity, but many factors have been proposed, including scavenging of cellular debris in expanding adipose tissue. It is known that MSR1 levels do not change during lipid infusion‐induced insulin resistance (Kashyap et al. 2009), and it remains possible that changes in MSR1 levels simply represent changes in macrophage numbers in the adipose tissue during obesity, since this is the cell type that predominantly expresses MSR1.

Previous work shrewdly identified that lysophosphatidylcholine was an obesity‐relevant ligand that engaged MSR1 to promote adipose tissue resident macrophage polarization away from inflammatory phenotypes (Zhu et al. 2014). We tested if a naturally occurring environmental ligand of MSR1, fucoidan, altered inflammation and/or glycemia in obese mice. Fucoidan is a sulphated polysaccharide found in brown seaweeds and is used as a dietary supplement. Fucoidan has been shown to lower blood glucose, insulin resistance, steatosis, ER stress, and inflammation in obese mice and rats (Jeong et al. 2013; Wang et al. 2016a,b), which supported our initial hypothesis that fucoidan would improve glycemia in obese mice and that MSR1 would mitigate its efficacy. Surprisingly, fucoidan worsened insulin resistance in an MSR1‐dependent manner. We found no overt change in markers of adipose tissue inflammation or ER stress due to fucoidan treatment or *Msr1* deletion. This was surprising given the well‐known role of MSR1 in immunity. It is not clear how deletion of this scavenger receptor reduces the ability of insulin to promote glucose uptake into adipose tissue. It is likely that reduced glucose uptake in adipose tissue of *Msr1* ^−/−^ mice is due to adipocyte insulin resistance. Future experiments defining if MSR1 alters local signals beyond inflammation such as adipokines and how this impacts steps in the insulin signaling cascade are warranted.

Previous studies that showed improved glucose control with fucoidan treatment used higher doses of this compound (80–100 mg/kg), whereas our study used 40 mg/kg. This could explain discordant results, but we propose that the most important advance provided in our study is the ligand–receptor specificity of the actions of fucoidan on MSR1. Sulphated polysaccharides have many potential cellular targets that depend on the dose–response relationships and, to the best of our knowledge, no ligand–receptor relationship has been established for the actions of fucoidan on blood glucose. This is an important consideration since human work supports the concept for fucoidan worsening insulin resistance (Hernández‐Corona et al. 2014). In fact, a daily oral dose of 500 mg of fucoidan to overweight/obese humans for 3 months increased both insulin levels and a marker of insulin resistance (HOMA‐IR). This fucoidan‐mediated deterioration of insulin resistance in obese humans occurred despite a reduction in blood pressure and lowering of LDL cholesterol (Hernández‐Corona et al. 2014). This warrants investigation of the role of MSR1 in glucose metabolism versus cardiovascular disease. Our results in mice highlight the need for caution in targeting immune responses to reduce metabolic inflammation or insulin resistance. A better understanding of ligand–receptor relationships relevant to glucose control should help inform immunometabolic approaches aiming to treat and/or prevent metabolic diseases.

## Conflict of Interest

The authors declare that they have no competing interests.

## References

[phy213930-bib-0001] Arredouani, M. , Z. Yang , Y. Ning , G. Qin , R. Soininen , K. Tryggvason , et al. 2004 The scavenger receptor MARCO is required for lung defense against pneumococcal pneumonia and inhaled particles. J. Exp. Med. 200:267–272. 10.1084/jem.20040731 15263032 PMC2212010

[phy213930-bib-0002] Babitt, J. , B. Trigatti , A. Rigotti , E. J. Smart , R. G. Anderson , S. Xu , et al. 1997 Murine SR‐BI, a high density lipoprotein receptor that mediates selective lipid uptake, is N‐glycosylated and fatty acylated and colocalizes with plasma membrane caveolae. J. Biol. Chem. 272:13242–13249. 10.1074/jbc.272.20.13242 9148942

[phy213930-bib-0003] Bauernfeind, F. , S. Niepmann , P. A. Knolle , and V. Hornung . 2016 Aging‐associated TNF production primes inflammasome activation and NLRP3‐related metabolic disturbances. J. Immunol. 197:2900–2908. 10.4049/jimmunol.1501336 27566828

[phy213930-bib-0004] Ben, J. , X. Zhu , H. Zhang , and Q. Chen . 2015 Class A1 scavenger receptors in cardiovascular diseases. Br. J. Pharmacol. 172:5523–5530. 10.1111/bph.13105. 25651870 PMC4667868

[phy213930-bib-0005] Bowdish, D. M. E. , and S. Gordon . 2009 Conserved domains of the class A scavenger receptors: evolution and function. Immunol. Rev. 227:19–31. 10.1111/j.1600-065X.2008.00728.x. 19120472

[phy213930-bib-0006] Caesar, R. , V. Tremaroli , P. Kovatcheva‐Datchary , P. D. Cani , and F. Bäckhed . 2015 Crosstalk between gut microbiota and dietary lipids aggravates WAT inflammation through TLR signaling. Cell Metab. 22:658–668. 10.1016/j.cmet.2015.07.026. 26321659 PMC4598654

[phy213930-bib-0007] Cani, P. D. , J. Amar , M. A. Iglesias , M. Poggi , C. Knauf , D. Bastelica , et al. 2007 Metabolic endotoxemia initiates obesity and insulin resistance. Diabetes 56:1761–1772. 10.2337/db06-1491. 17456850

[phy213930-bib-0008] Cavallari, J. F. , M. D. Fullerton , B. M. Duggan , K. P. Foley , E. Denou , B. K. Smith , et al. 2017 Muramyl dipeptide‐based postbiotics mitigate obesity‐induced insulin resistance via IRF4. Cell Metab. 25:1063–1074.e3. 10.1016/j.cmet.2017.03.021. 28434881

[phy213930-bib-0009] Chi, W. , D. Dao , T. C. Lau , B. D. Henriksbo , J. F. Cavallari , K. P. Foley , et al. 2014 Bacterial peptidoglycan stimulates adipocyte lipolysis via NOD1. PLoS ONE 9:e97675 10.1371/journal.pone.0097675. 24828250 PMC4020832

[phy213930-bib-0010] Denou, E. , K. Lolmède , L. Garidou , C. Pomie , C. Chabo , T. C. Lau , et al. 2015 Defective NOD2 peptidoglycan sensing promotes diet‐induced inflammation, dysbiosis, and insulin resistance. EMBO Mol. Med. 7:259–274. 10.15252/emmm.201404169. 25666722 PMC4364944

[phy213930-bib-0011] Dorrington, M. G. , A. M. Roche , S. E. Chauvin , Z. Tu , K. L. Mossman , J. N. Weiser , et al. 2013 MARCO is required for TLR2‐ and Nod2‐mediated responses to *Streptococcus pneumoniae* and clearance of pneumococcal colonization in the murine nasopharynx. J. Immunol. 190:250–258. 10.4049/jimmunol.1202113. 23197261 PMC3529821

[phy213930-bib-0012] Ehses, J. A. , D. T. Meier , S. Wueest , J. Rytka , S. Boller , P. Y. Wielinga , et al. 2010 Toll‐like receptor 2‐deficient mice are protected from insulin resistance and beta cell dysfunction induced by a high‐fat diet. Diabetologia 53:1795–1806. 10.1007/s00125-010-1747-3. 20407745

[phy213930-bib-0013] Elbein, S. C. , P. A. Kern , N. Rasouli , A. Yao‐Borengasser , N. K. Sharma , and S. K. Das . 2011 Global gene expression profiles of subcutaneous adipose and muscle from glucose‐tolerant, insulin‐sensitive, and insulin‐resistant individuals matched for BMI. Diabetes 60:1019–1029. 10.2337/db10-1270. 21266331 PMC3046820

[phy213930-bib-0014] Febbraio, M. , N. A. Abumrad , D. P. Hajjar , K. Sharma , W. Cheng , S. F. Pearce , et al. 1999 A null mutation in murine CD36 reveals an important role in fatty acid and lipoprotein metabolism. J. Biol. Chem. 274:19055–19062. 10.1074/JBC.274.27.19055. 10383407

[phy213930-bib-0015] Goudriaan, J. R. , V. E. H. Dahlmans , B. Teusink , D. M. Ouwens , M. Febbraio , J. A. Maassen , et al. 2003 CD36 deficiency increases insulin sensitivity in muscle, but induces insulin resistance in the liver in mice. J. Lipid Res. 44:2270–2277. 10.1194/jlr.M300143-JLR200. 12923231

[phy213930-bib-0016] Henriksbo, B. D. , and J. D. Schertzer . 2015 Is immunity a mechanism contributing to statin‐induced diabetes? Adipocyte 4:232–238. 10.1080/21623945.2015.1024394. 26451278 PMC4573193

[phy213930-bib-0017] Henriksbo, B. D. , T. C. Lau , J. F. Cavallari , E. Denou , W. Chi , J. S. Lally , et al. 2014 Fluvastatin causes NLRP3 inflammasome‐mediated adipose insulin resistance. Diabetes 63:3742–3747. 10.2337/db13-1398. 24917577

[phy213930-bib-0018] Hernández‐Corona, D. M. , E. Martínez‐Abundis , and M. González‐Ortiz . 2014 Effect of fucoidan administration on insulin secretion and insulin resistance in overweight or obese adults. J. Med. Food 17:830–832. 10.1089/jmf.2013.0053. 24611906

[phy213930-bib-0019] Hotamisligil, G. S. , N. S. Shargill , and B. M. Spiegelman . 1993 Adipose expression of tumor necrosis factor‐alpha: direct role in obesity‐linked insulin resistance. Science 259:87–91. 7678183 10.1126/science.7678183

[phy213930-bib-0020] Hotamisligil, G. S. , P. Arner , J. F. Caro , R. L. Atkinson , and B. M. Spiegelman . 1995 Increased adipose tissue expression of tumor necrosis factor‐alpha in human obesity and insulin resistance. J. Clin. Investig. 95:2409–2415. 10.1172/JCI117936. 7738205 PMC295872

[phy213930-bib-0021] Jeong, Y.‐T. , Y. D. Kim , Y.‐M. Jung , D.‐C. Park , D.‐S. Lee , S.‐K. Ku , et al. 2013 Low molecular weight fucoidan improves endoplasmic reticulum stress‐reduced insulin sensitivity through AMP‐activated protein kinase activation in L6 myotubes and restores lipid homeostasis in a mouse model of type 2 diabetes. Mol. Pharmacol. 84:147–157. 10.1124/mol.113.085100. 23658008

[phy213930-bib-0022] Jorgensen, S. B. , H. M. O'Neill , L. Sylow , J. Honeyman , K. A. Hewitt , R. Palanivel , et al. 2013 Deletion of skeletal muscle SOCS3 prevents insulin resistance in obesity. Diabetes 62:56–64. 10.2337/db12-0443. 22961088 PMC3526029

[phy213930-bib-0023] Kashyap, S. R. , A. G. Ioachimescu , H. L. Gornik , T. Gopan , M. B. Davidson , A. Makdissi , et al. 2009 Lipid‐induced insulin resistance is associated with increased monocyte expression of scavenger receptor CD36 and internalization of oxidized LDL. Obesity 17:2142–2148. 10.1038/oby.2009.179. 19521352 PMC2836489

[phy213930-bib-0024] Lancaster, G. I. , K. G. Langley , N. A. Berglund , H. L. Kammoun , S. Reibe , E. Estevez , et al. 2018 Evidence that TLR4 is not a receptor for saturated fatty acids but mediates lipid‐induced inflammation by reprogramming macrophage metabolism. Cell Metab. 27:1096–1110.e5. 10.1016/j.cmet.2018.03.014. 29681442

[phy213930-bib-0025] Liu, P.‐S. , H. Wang , X. Li , T. Chao , T. Teav , S. Christen , et al. 2017 *α*‐ketoglutarate orchestrates macrophage activation through metabolic and epigenetic reprogramming. Nat. Immunol. 18:985–994. 10.1038/ni.3796. 28714978

[phy213930-bib-0026] McBride, M. J. , K. P. Foley , D. M. D'Souza , Y. E. Li , T. C. Lau , T. J. Hawke , et al. 2017 The NLRP3 inflammasome contributes to sarcopenia and lower muscle glycolytic potential in old mice. Am. J. Physiol. Endocrinol. Metab. 313:E222–E232. 10.1152/ajpendo.00060.2017. 28536183 PMC5582883

[phy213930-bib-0027] McPhee, J. B. , and J. D. Schertzer . 2015 Immunometabolism of obesity and diabetes: microbiota link compartmentalized immunity in the gut to metabolic tissue inflammation. Clin. Sci. 129:1083–1096. 10.1042/CS20150431. 26464517

[phy213930-bib-0028] Mills, E. L. , B. Kelly , A. Logan , A. S. H. Costa , M. Varma , C. E. Bryant , et al. 2016 Succinate dehydrogenase supports metabolic repurposing of mitochondria to drive inflammatory macrophages. Cell 167:457–470.e13. 10.1016/j.cell.2016.08.064. 27667687 PMC5863951

[phy213930-bib-0029] Oliveira, M. C. , Z. Menezes‐Garcia , M. C. C. Henriques , F. M. Soriani , V. Pinho , A. M. C. Faria , et al. 2013 Acute and sustained inflammation and metabolic dysfunction induced by high refined carbohydrate‐containing diet in mice. Obesity 21:E396–E406. 10.1002/oby.20230. 23696431

[phy213930-bib-0030] Park, Y. M. , S. R. Kashyap , J. A. Major , and R. L. Silverstein . 2012 Insulin promotes macrophage foam cell formation: potential implications in diabetes‐related atherosclerosis. Lab. Invest. 92:1171–1180. 10.1038/labinvest.2012.74. 22525426 PMC3407326

[phy213930-bib-0031] Parthasarathy, S. , D. J. Printz , D. Boyd , L. Joy , and D. Steinberg . 1986 Macrophage oxidation of low density lipoprotein generates a modified form recognized by the scavenger receptor. Arteriosclerosis 6:505–510. 10.1161/01.atv.6.5.505 3767695

[phy213930-bib-0032] Pestana, D. , D. Teixeira , M. Meireles , C. Marques , S. Norberto , C. Sá , et al. 2017 Adipose tissue dysfunction as a central mechanism leading to dysmetabolic obesity triggered by chronic exposure to p, p’‐DDE. Sci. Rep. 7:2738 10.1038/s41598-017-02885-9. 28572628 PMC5453948

[phy213930-bib-0033] Schertzer, J. D. , A. K. Tamrakar , J. G. Magalhães , S. Pereira , P. J. Bilan , M. D. Fullerton , et al. 2011 NOD1 activators link innate immunity to insulin resistance. Diabetes 60:2206–2215. 10.2337/db11-0004. 21715553 PMC3161332

[phy213930-bib-0034] Shi, H. , M. V. Kokoeva , K. Inouye , I. Tzameli , H. Yin , and J. S. Flier . 2006 TLR4 links innate immunity and fatty acid‐induced insulin resistance. J. Clin. Investig. 116:3015–3025. 10.1172/JCI28898. 17053832 PMC1616196

[phy213930-bib-0035] Suzuki, H. , Y. Kurihara , M. Takeya , N. Kamada , M. Kataoka , K. Jishage , et al. 1997 A role for macrophage scavenger receptors in atherosclerosis and susceptibility to infection. Nature 386:292–296. 10.1038/386292a0. 9069289

[phy213930-bib-0036] Vandanmagsar, B. , Y.‐H. Youm , A. Ravussin , J. E. Galgani , K. Stadler , R. L. Mynatt , et al. 2011 The NLRP3 inflammasome instigates obesity‐induced inflammation and insulin resistance. Nat. Med. 17:179–188. 10.1038/nm.2279. 21217695 PMC3076025

[phy213930-bib-0037] Wang, Y. , J. Wang , Y. Zhao , S. Hu , D. Shi , and C. Xue . 2016a Fucoidan from sea cucumber *Cucumaria frondosa* exhibits anti‐hyperglycemic effects in insulin resistant mice via activating the PI3K/PKB pathway and GLUT4. J. Biosci. Bioeng. 121:36–42. 10.1016/j.jbiosc.2015.05.012. 26194305

[phy213930-bib-0038] Wang, J. , S. Hu , W. Jiang , W. Song , L. Cai , and J. Wang . 2016b Fucoidan from sea cucumber may improve hepatic inflammatory response and insulin resistance in mice. Int. Immunopharmacol. 31:15–23. 10.1016/j.intimp.2015.12.009. 26690975

[phy213930-bib-0039] Weisberg, S. P. , D. McCann , M. Desai , M. Rosenbaum , R. L. Leibel , and A. W. Ferrante . 2003 Obesity is associated with macrophage accumulation in adipose tissue. J. Clin. Investig. 112:1796–1808. 10.1172/JCI19246. 14679176 PMC296995

[phy213930-bib-0040] Wellen, K. E. , and G. S. Hotamisligil . 2005 Inflammation, stress, and diabetes. J. Clin. Investig. 115:1111–1119. 10.1172/JCI25102. 15864338 PMC1087185

[phy213930-bib-0041] Wilson, C. G. , J. L. Tran , D. M. Erion , N. B. Vera , M. Febbraio , and E. J. Weiss . 2016 Hepatocyte‐specific disruption of CD36 attenuates fatty liver and improves insulin sensitivity in HFD‐fed mice. Endocrinology 157:570–585. 10.1210/en.2015-1866. 26650570 PMC4733118

[phy213930-bib-0042] Winer, S. , and D. A. Winer . 2012 The adaptive immune system as a fundamental regulator of adipose tissue inflammation and insulin resistance. Immunol. Cell Biol. 90:755–762. 10.1038/icb.2011.110. 22231651

[phy213930-bib-0043] Winer, D. A. , H. Luck , S. Tsai , and S. Winer . 2016 The intestinal immune system in obesity and insulin resistance. Cell Metab. 23:413–426. 10.1016/j.cmet.2016.01.003. 26853748

[phy213930-bib-0044] Xu, H. , G. T. Barnes , Q. Yang , G. Tan , D. Yang , C. J. Chou , et al. 2003 Chronic inflammation in fat plays a crucial role in the development of obesity‐related insulin resistance. J. Clin. Investig. 112:1821–1830. 10.1172/JCI19451. 14679177 PMC296998

[phy213930-bib-0045] Zani, I. A. , S. L. Stephen , N. A. Mughal , D. Russell , S. Homer‐Vanniasinkam , S. B. Wheatcroft , et al. 2015 Scavenger receptor structure and function in health and disease. Cells 4:178–201. 10.3390/cells4020178. 26010753 PMC4493455

[phy213930-bib-0046] Zhu, X. , G. Zong , L. Zhu , Y. Jiang , K. Ma , H. Zhang , et al. 2014 Deletion of class A scavenger receptor deteriorates obesity‐induced insulin resistance in adipose tissue. Diabetes 63:562–577. 10.2337/db13-0815. 24170693

